# Meta-analysis comparing the efficacy of nedaplatin-based regimens between squamous cell and non-squamous cell lung cancers

**DOI:** 10.18632/oncotarget.17499

**Published:** 2017-04-28

**Authors:** Yijun Tian, Qian Liu, Kongju Wu, Qian Chu, Yuan Chen, Kongming Wu

**Affiliations:** ^1^ Department of Oncology, Tongji Hospital of Tongji Medical College, Huazhong University of Science and Technology, Wuhan, P.R. China; ^2^ Medical School of Pingdingshan University, Pingdingshan, P.R. China

**Keywords:** nedaplatin, non-small cell lung cancer, squamous cell lung cancer, chemotherapy

## Abstract

Non-small cell lung cancer (NSCLC) consists of several subtypes, including adenocarcinoma, squamous cell lung cancer, large cell lung cancer, and other rarer types. Platinum-based regimens are currently the standard for treatment of advanced NSCLC. Nedaplatin is reportedly associated with a high response rate in squamous cell lung cancer. However, the relevant studies are small and mainly descriptive. The purpose of this meta-analysis was therefore to compare the efficacy of nedaplatin in squamous cell lung cancer with that in non-squamous cell lung cancer. Studies concerning nedaplatin-based regimens in NSCLC patients were retrieved from PubMed and EMBASE. The response rate for nedaplatin-based regimens in squamous cell lung cancer (ORR: 55.6%, 95% CI: 52.5-58.7%) was higher (OR: 1.55, 95% CI: 1.17-2.05) than that for non-squamous cell lung cancer (ORR: 34.4%, 95% CI: 32.3-36.5%). In addition, Taxane plus nedaplatin produced a longer overall and progress-free survival than CPT-11 or gemcitabine plus nedaplatin. To verify these findings, future well-controlled clinical studies will be needed.

## INTRODUCTION

Most of non-small cell lung cancer (NSCLC) patients are diagnosed at an advanced stage and have lost their chance for surgery. Consequently, NSCLC has the highest cancer mortality rates in both males and females [1]. NSCLC consists of several subtypes, including adenocarcinoma, squamous cell lung cancer, large cell lung cancer, and other rarer types. Although the discoveries of driver-mutation and personalized medications have improved the prognosis of a portion of NSCLC patients [2], platinum-based chemotherapies still serve as the first-line treatments for advanced NSCLC patients who are unsuitable for targeted therapies. In a study of 1139 patients, platinum-based regimens all had similar efficacies, with a response rate of nearly 19% and a median overall survival time of 7.9 months [3]. To improve efficacy and avoid side effects, nedaplatin was synthesized and introduced into clinical trials in 1988. Preliminary results indicated that nedaplatin might be an excellent substitute for cisplatin [4]. Subsequent research demonstrated that nedaplatin-based regimens have favorable efficacy in squamous cell histology. Naito [5] investigated efficacy of a nedaplatin and docetaxel regimen in advanced squamous cell lung carcinoma. The doublet produced an objective response rate of 62% and an overall survival of 16.1 months, which was comparable to the results in earlier studies [6, 7]. But although attention has been paid to the efficacy of nedaplatin and its comparison to cisplatin, few studies have focused on its histological specificity. This systemic review was therefore designed to quantitatively compare the efficacy of a nedaplatin-based regimen between squamous cell lung cancer and non-squamous cell lung cancer.

## RESULTS

### Nedaplatin-based regimens are more effective against squamous cell lung cancer than non-squamous cell lung cancer

**Figure 1:**
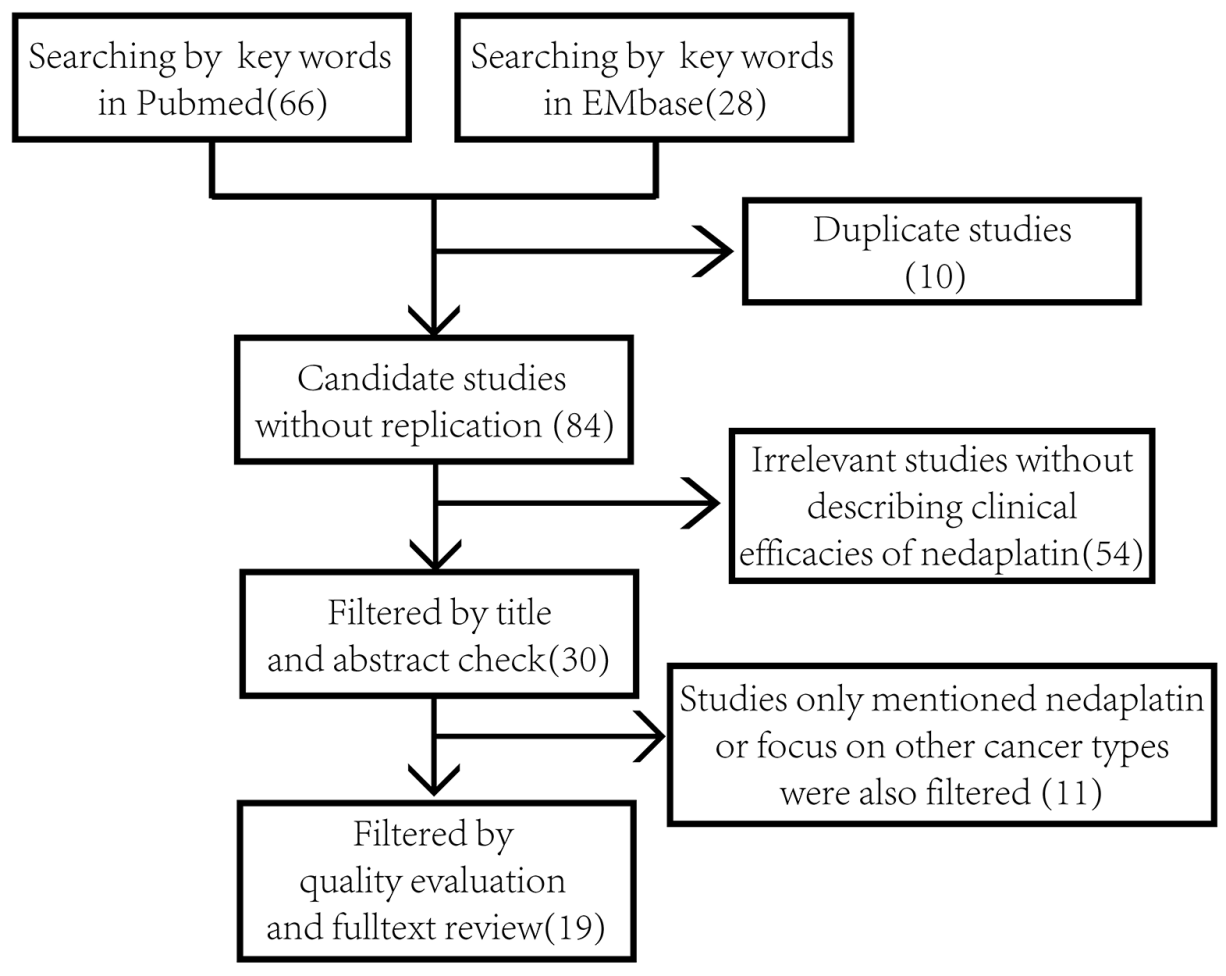
Flow diagram of the process of literature retrieval and filtering Numbers in the brackets refer to the sum of studies after each part of the screening.

Details of the process used to screen the studies are listed in Figure 1. The characteristics of the included studies are presented in Table 1. Of the 19 included studies, 8 assessed effects using WHO criteria, while 11 used RECIST criteria. The regimens applied in these studies include: Irinotecan + nedaplatin (4 studies), gemcitabine + nedaplatin (5 studies), taxane + nedaplatin (6 studies), S-1 + nedaplatin (1 study), nedaplatin single agent (2 studies), and a mixed doublet regimen (1 study). The clinical responses to respective treatment regimens were defined as complete or partial according to criteria applied in each study. After reviewing the full texts, we included 12 studies covering a total of 867 patients describing the efficacies of nedaplatin-containing regimens in squamous cell lung cancer and non-squamous cell lung cancer. We also reviewed the eligibility criteria for each study and found that only one study focused on the efficacy in elderly people [13]. Other general inclusion criteria are described in detail in the method section. Five studies included not-naïve-to-chemotherapy patients. We inspected each study and found that 4 of them declared no difference in response rate between previously untreated and treated patients [9, 12, 16, 18]. The remaining study included one recurrent patient after chemotherapy [8]. There was no difference in gender distributions in the included cases.

**Table 1:** Characteristics of the included studies

First Author	Phase	Regime Intensity (mg/m^2^)	Chemo Naive	SQC	Non-SQC	Effect Evaluation Criteria	Common Hematologic Toxicity	Common Non-Hematologic Toxicity	Overall Survival	Progress-free Survival
Oshita (2003) [7]	I/II	CPT (60) + Nedaplatin (50-100)	No	8	30	WHO	Neutropenia Anemia	GPT Elevation	11.4 ms	-
Kurata (2004) [8]	I	GEM (800-1000) + Nedaplatin (60-100)	No	7	13	RECIST	Neutropenia Thrombocytopenia	Transaminase Elevation Nausea Appetite loss	9.1 ms	5.1 ms
Oshita (2004) [9]	II	CPT (60) + Nedaplatin (100)	Yes	11	26	RECIST	Neutropenia Anemia Thrombocytopenia	Diarrhea Nausea	13.9 ms	-
Shirai (2006) [10]	II	GEM (1000) + Nedaplatin (100)	Yes	4	29	WHO	Neutropenia Thrombocytopenia Anemia	Transaminase Elevation Nausea	9.0 ms	4.9 ms
Oshita (2011) [11]	II	CPT (50-60) + Nedaplatin (60)	No	12	38	RECIST	Febrile neutropenia	Diarrhea	14.5 ms vs 9.1 ms	-
Yamamoto (2009) [12]	I	Nedaplatin (60-100)	Yes	21	18	WHO	Neutropenia	Nausea Anorexia	11.2 ms	-
Teramoto (2012) [13]	II	DOC (60) + Nedaplatin (80)	Yes	12	29	RECIST	Neutropenia	Nausea Anorexia	13.0 ms	7.4 ms
Yang (2012) [14]	RCT	GEM (1250) + Nedaplatin (80)	Yes	3	21	RECIST	Neutropenia Anemia	Nausea	17.5 ms	6 ms
Li (2014) [15]	Retrospective study	PEM (500), Doc (75), GEM (1000), NVB (25), PAX (175) + Nedaplatin (75)	No	103	191	WHO	Neutropenia	Indirect bilirubin elevation, Nausea, Transaminase Elevation	14.7 ms	-
Zhang (2014) [16]	Retrospective study	Pac (175), Doc (75) + Nedaplatin (80)	Yes	63	149	RECIST	Neutropenia	NS	18.5 ms	10.4 ms
Fukuda (1990) [17]	II	Nedaplatin (100)	No	9	52	WHO	Neutropenia Anemia	Nausea	-	-
Sekine (2007) [18]	I	PAX (120-150) + Nedaplatin (80) + DCRT (60Gy/30F)	Yes	6	12	RECIST	Neutropenia	Pneumonitis	-	9.7 ms
Yamada (2015)[19]	II	CPT (60) + Nedaplatin (100)	Yes	50	-	RECIST	Neutropenia Anemia	Anoxia	11.8 ms	4.3 ms
Shukuya (2015)[20]	III	DOC (60) + Nedaplatin (100)	No	172	-	RECIST	Neutropenia	Nausea Anorexia	13.6 ms	4.9 ms
Naito (2011) [5]	II	DOC (60) + Nedaplatin (100)	Yes	21	-	RECIST	Neutropenia	Nausea Anorexia Diarrhea	16.1 ms	7.4 ms
Masago (2011) [21]	I	GEM (800-1000) + Nedaplatin (70-80)	Yes	13	-	RECIST	Neutropenia Anemia	Nausea Anorexia	10.5 ms	-
Sekine (2004) [6]	I	PAX (135-210) + Nedaplatin (60-100)	Yes	42	-	WHO	Neutropenia	Infection	11.1 ms	-
Hirose (2003) [22]	I	GEM (800-1000) + Nedaplatin (60-100)	Yes	-	20	WHO	Neutropenia Anemia	Nausea Transaminase Elevation	8.0 ms	5.0 ms
Tang (2014) [23]	Comparison Study	S-1 (40-60) + Nedaplatin	No	-	/91	WHO	Neutropenia Anemia Thrombocytopenia	Nausea	-	3.34 ms

*SQC, Squamous cell lung cancer; CPT, Irinotecan; GEM, Gemcitabine; DOC, Docetaxel; PEM, Pemetrexed; NVB, PAX, Paclitaxel; DCRT, 3-Dimensional conformal radiotherapy; RECIST, Response Evaluation Criteria In Solid Tumors; WHO, World Health Organization evaluation criteria; GPT, glutamic-pyruvic transaminase; ms, months.

**Figure 2:**
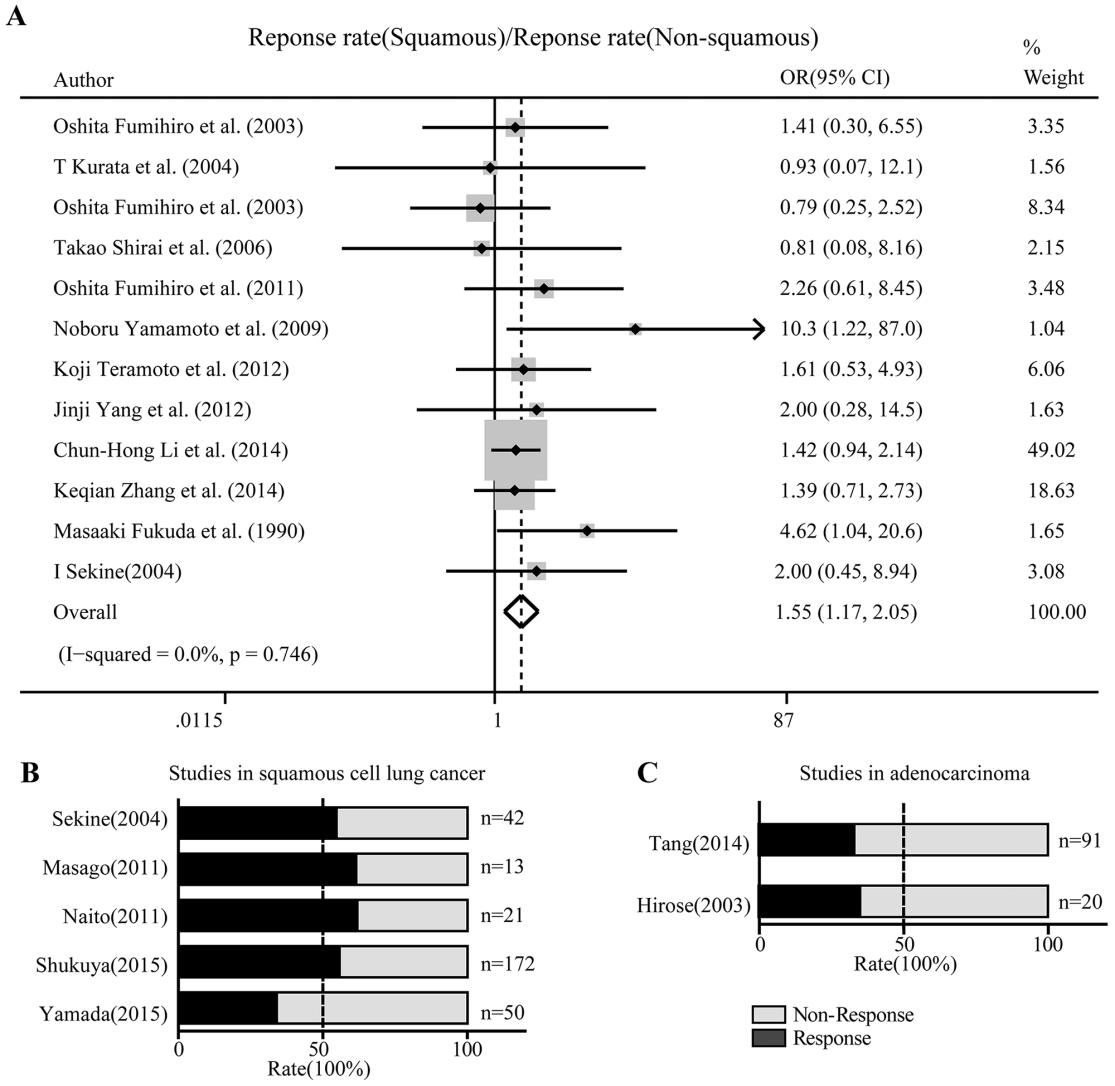
Nedaplatin-based schemes showed superior activity against squamous cell lung cancer than non-squamous cell lung cancer **(A)** Forest plot in which the ORs of the response rates between squamous cell and non-squamous cell cancers were compared using a fixed effect model. OR, odds ratio; CI: confidential interval. **(B)** Proportional histogram of studies describing the responsive and non-responsive fractions among squamous cell lung cancer patients. **(C)** Proportional histogram of studies describing the responsive and non-responsive fractions among lung adenocarcinoma patients. Responses to the respective treatment regimens were defined as complete or partial.

The overall odds ratio (OR) was defined as the response rate in squamous cell lung cancer over that in non-squamous cell lung cancer, which was 1.55 with 95% CI: 1.17-2.05 (Figure 2A). The overall response rate was 55.6% (95% CI: 52.5-58.7%) in squamous cell lung cancer and 34.4% (95% CI: 32.3-36.5%) in non-squamous cell lung cancer. Age, gender and pre-chemo status did not correlate with response rate.

### Other characteristics of nedaplatin-based regimens in non-small cell lung cancer

**Figure 3:**
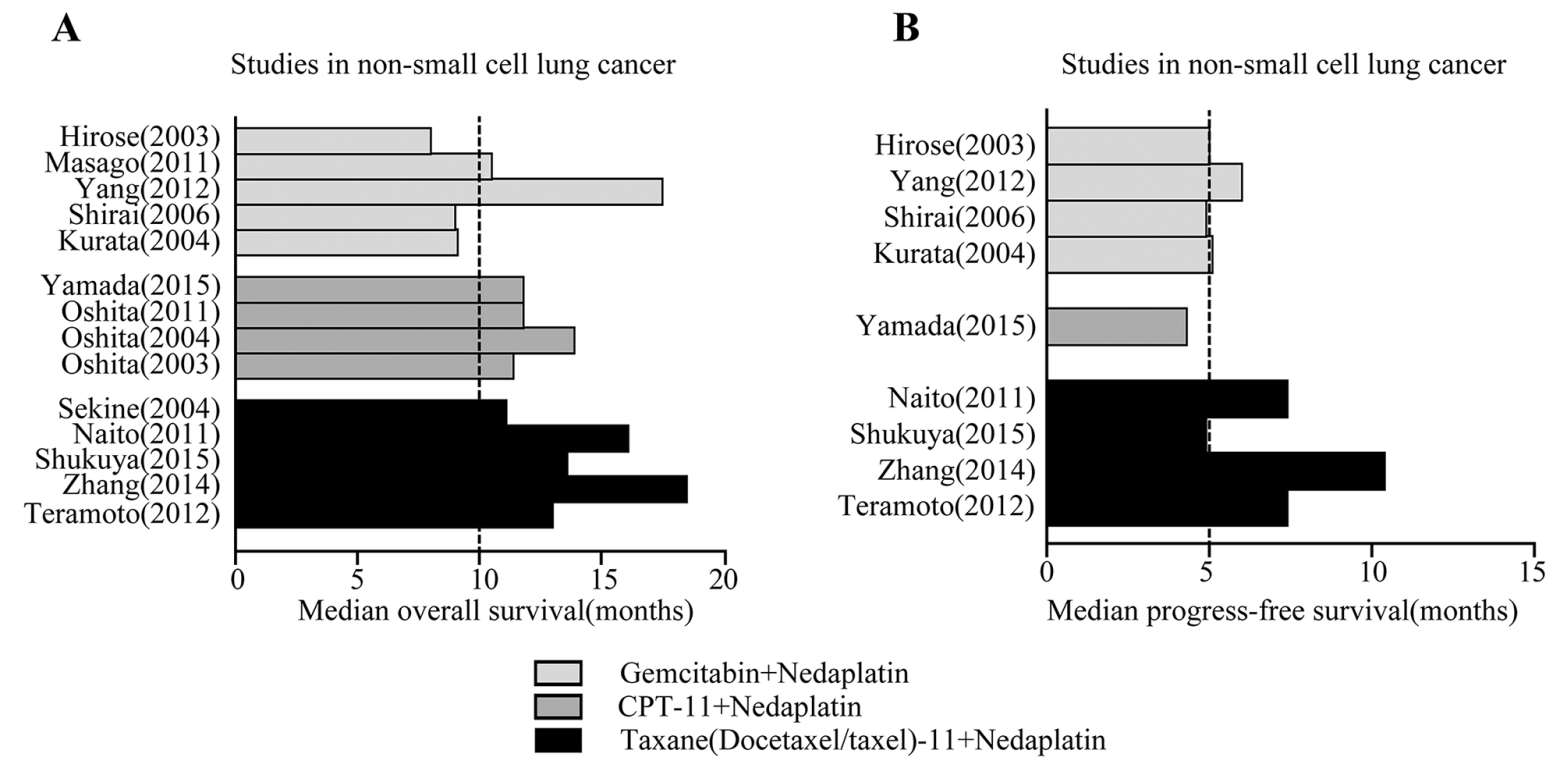
Prognosis after Nedaplatin-based chemotherapy Comparison of overall survival **(A)** and progress free survival **(B)** for gemcitabine (GEM), irinotecan (CPT-11) or taxane (docetaxel or paclitaxel) plus nedaplatin in NSCLC.

For studies describing the response rate only in squamous cell lung cancer or in non-squamous cell lung cancer, we present the response fractions in the respective studies by plotting a proportional histogram. Four of the five studies on squamous cell lung cancer showed a response rate over 50% (Figure 2B), while two studies reached about 30-40% for non-squamous cell lung cancer (Figure 2C). In addition to the response rate to nedaplatin-based regimens in squamous cell and non-squamous cell lung cancers, we also recorded other fundamental characteristics of the regimens. In the 19 included studies, common hematologic toxicities were neutropenia, thrombocytopenia and anemia, while the common non-hematologic toxicities were nausea, anorexia and transaminase elevation. With respect to overall survival, we found that among three common nedaplatin-based regimens in 5 studies, taxane (docetaxel or paclitaxel) plus nedaplatin was associated with a median overall survival time of at least 10 months (Figure 3A) and a median progress-free survival time of at least 5 months (Figure 3B). Regimens contain CPT-11 and gemcitabine with nedaplatin produced shorter overall survival and progress-free survival.

### Publication bias and sensitivity analysis

**Figure 4:**
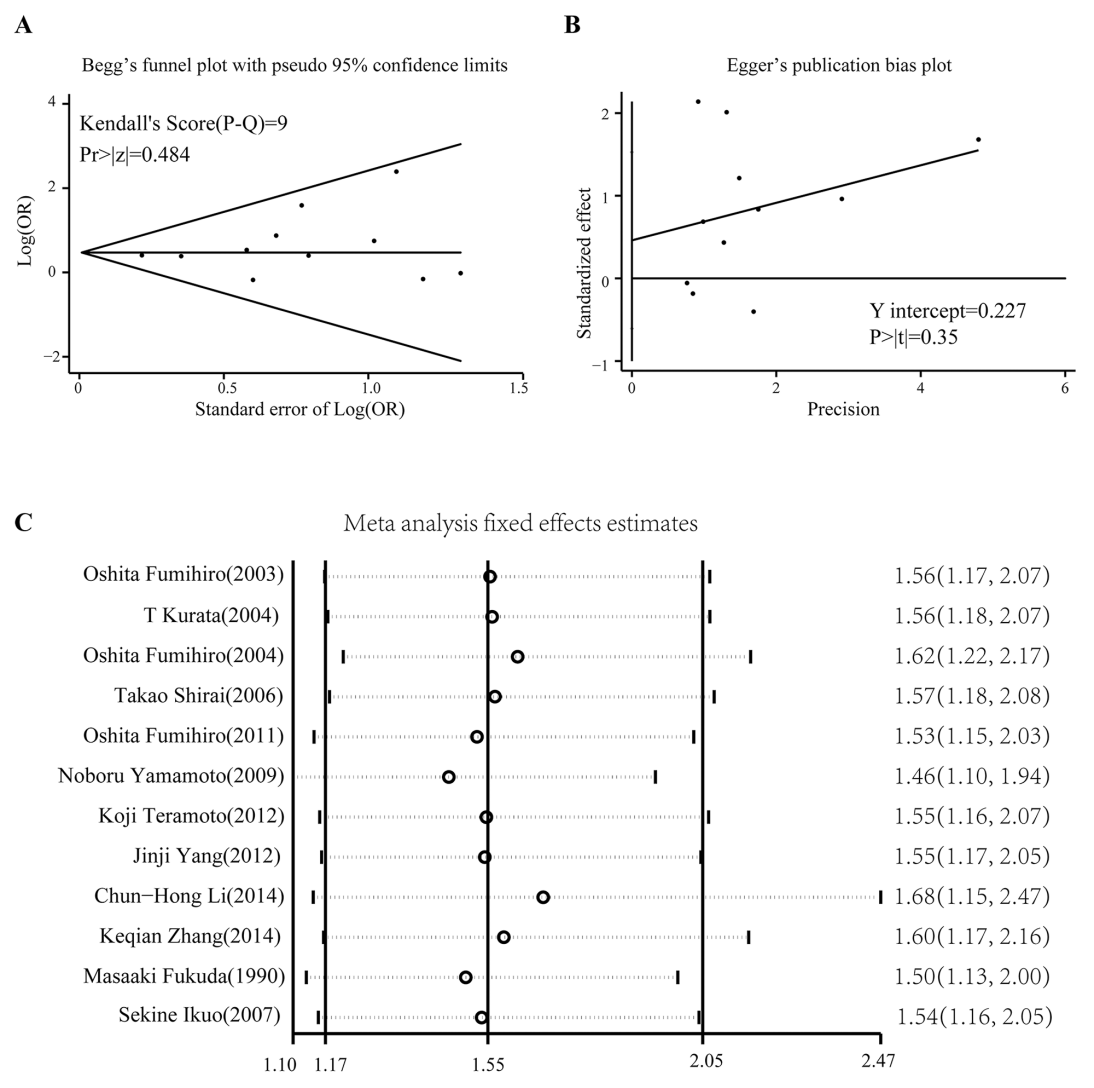
Publication bias test and sensitivity analysis **(A)** Begg’s funnel plot for detection of possible publication bias. The limits refer to the pseudo 95% confidence interval. **(B)** Egger’s publication bias plot also reveals no publication bias. **(C)** Effect of each study on the overall OR analyzed using a recessive model (squamous cell lung cancer vs non-squamous cell lung cancer).

Begg’s test and Egger’s test were used to evaluate publication bias. No significant biases were found in our analysis: Begg’s test, p = 0.533; Egger’s test, p = 0.354. According to the funnel plot in Begg’s test, all studies were within the 95% confidence limitations (Figure 4A). Another Egger’s publication bias plot also suggested little bias among included studies (Figure 4B). The heterogeneity test yielded an I^2^ of 0.0% and p value of 0.746, which indicates little heterogeneity across the included studies. Therefore, the fixed effects model was introduced in the statistics. We also conducted a sensitivity analysis using the metainf command in STATA 12.0. Omitting any one study did not significantly change the pooled OR value (Figure 4C).

## DISCUSSION

Developed in 1983, nedaplatin is a second-generation platinum compound that features minor nephrotoxicity and neurotoxicity [24]. Moreover, the antitumor activity of nedaplatin may be mediated by mechanisms different from p53-dependent early apoptosis [25]. According to a phase II study, nedaplatin is more active against squamous cell lung cancer than lung adenocarcinoma [7]. However, the histologic differences have not been fully identified and investigated as a primary interest. Our analysis in the present study confirmed that nedaplatin-based regimens show better anti-tumor activity against squamous cell than non-squamous cell lung cancers. Administration of nedaplatin produced a higher concentration of platinum atom in squamous cell lung cancer than adenocarcinoma. In addition, we recently observed that in a NSCLC cell line, nedaplatin activity was less determined by NRF2 (nuclear factor, erythroid 2 like 2) signaling than was cisplatin [26]. Considering that over-activation of NRF2 is more frequent in squamous cell than non-squamous cell lung cancers, and NRF2 is involved in resistance to chemotherapy and kinase targeted therapy [27, 28], this difference may account for superior efficacy of nedaplatin against squamous cell lung cancer.

It should be noted that no studies directly focused on prognosis after nedaplatin treatment in squamous cell and non-squamous cell lung cancers. The available studies indicate CPT-11 or taxane plus nedaplatin regimens were associated with longer overall and progress-free survival than gemcitabine plus nedaplatin in NSCLC. Early research found that the combination of nedaplatin plus CPT-11 or paclitaxel produced synergistic antitumor activity in a sequence-dependent manner. Kanzawa et al [29] reported that concurrent administration of nedaplatin and CPT-11 produced a remarkably synergistic interaction in NSCLC and a small cell lung cancer cell line. Further analysis revealed a 10-fold enhancement of DNA topoisomerase I inhibition in the presence of μg/ml concentrations of nedaplatin. Another study showed that administrating paclitaxel prior to nedaplatin produced significantly stronger antitumor activity and lower body weight loss than nedaplatin prior to paclitaxel in a mouse xenograft model [30]. Because few studies compared survival between nedaplatin-based doublet regimens, we can only describe overall and progress-free survival for these studies. However, a multicenter phase III study reported that overall survival time for nedaplatin plus docetaxel (median: 13.6 months) was 2.2 months longer than for cisplatin plus docetaxel (median: 11.4 months) [20]. To confirm that nedaplatin produces a longer-term benefit in squamous cell lung cancer than non-squamous cell lung cancer, well-controlled head-to-head studies focusing on survival will be required.

This meta-analysis has several limitations. Subjects of the included studies were mostly from East Asia. Owing to health insurance policies and cost-effectiveness considerations, nedaplatin is seldom prescribed in western countries. Consequently, our conclusions will need to be reassessed in American or European patients. Although little heterogeneity was statistically observed, confounding factors may have affected each study. For example, whether nedaplatin was administered as monotherapy or combination therapy and differences between doublets would all affect the pooled OR. But considering that these factors were present in a minority studies and few studies met the requirements for pooled analysis, it is reasonable to perform this meta-analysis on nedaplatin-based treatment. To control for these confounding factors, a larger sample size and stratified analysis will be needed in future studies. A previous meta-analysis confirmed that nedaplatin achieved an overall response, overall survival, and survival probability equivalent to cisplatin in advanced NSCLC, but efficiency among different histology subtypes was not compared [31].

In sum, our meta-analysis indicates that nedaplatin-based regimens produced a better response rate in squamous cell than non-squamous cell lung cancers. Differences in toxicities of nedaplatin- and cisplatin-based regimens deserve physicians’ attention when treating NSCLC. Because different combinations of nedaplatin may influence the efficacy and patient survival, more clinical studies will be required in the future.

## MATERIALS AND METHODS

### Search strategy

We screened studies of nedaplatin-based treatments in advanced or relapsed NSCLC patients. To serve the purpose of our analysis, studies describing tumor responses to nedaplatin-based regimens in squamous cell and non-squamous cell lung cancers using the Response Evaluation Criteria in Solid Tumors (RECIST) and World Health Organization criterion (WHO) were included in the pooled analysis. Because there has been only limited research focusing on comparison of squamous cell with non-squamous cell lung cancer, we conducted our searching in a comprehensive manner. We searched PubMed by using terms “254-S OR Nedaplatin OR Aqupla AND Lung cancer”. An EMBASE search was performed using the terms “nedaplatin OR 254-S OR Aqupla AND Lung cancer”. Included were English-written randomized controlled trials (RCT), case-control and cohort studies published up to December 1, 2015. No area or district filters were added during the search. We also scanned clinicaltrials.gov for possible completed trials that had not yet been published. However, no relevant records were found. After deduplification, studies were subjected to title and abstract scans. Studies that did not describe the clinical efficacy of nedaplatin were removed (n=54). Through quality evaluation and fulltext review, studies that just mentioned nedaplatin or focused on other cancer types were also filtered (n=11). Twelve of the 19 studies describing tumor responses in squamous cell and non-squamous cell lung cancers were included in a pooled forest map, while those that described only the tumor response rate in the two sets were included in a proportional histogram. Other eligibility criteria included: 1) Eastern Cooperative Oncology Group (ECOG) performance status score no more than 3; 2) sufficient bone marrow function (white blood cell count at least 4,000/mm^3^ (μL) or absolute neutrophil count at least 2,000/mm^3^ (μL), platelet counts of at least 100,000/mm^3^ (μL), hemoglobin level of at least 9.0 g/dL and; 3) sufficient hepatic function (serum total bilirubin level no more than 1.5 mg/dL, serum aspartate aminotransferase and alanine aminotransferase level no more than 100 IU/L). Interest end points were objective response rate and disease control rate. Studies with poor quality or no interest endpoints were excluded.

### Data extraction

The following information was extracted from the included studies by two investigators: family name of the first author, year of publication, clinical trial stage (or type), doublet regimens applied, chemo-naive status of subject, numbers of squamous cell and non-squamous cell lung cancer patients whose responses were measurable, criteria used to evaluate efficacy of specific studies. Other characteristics such as common hematologic and non-hematologic toxicities, median overall survival, and progress-free survival were also recorded.

### Statistical analysis

Results were presented as ORs of the response rate in squamous cell lung cancer to the rate in non-squamous cell lung cancer. Heterogeneity among the ORs was assessed by applying the Cochran Q and I^2^ tests. A fixed effect model was used when I^2^ was less than 50%. Otherwise a random effects model was applied. The Egger regression asymmetry test was used to detect publication bias, characterized as p value less than 0.1. Pooled ORs were plotted as a forest map, and studies with only one arm were expressed by plotting a proportional histogram in GraphPad Prism 5. All statistical analyses were performed using the STATA software package (version 12.0) (Stata Corp Station, TX, USA).

## References

[1] Siegel RL, Miller K, Jemal A (2015). Cancer statistics, 2015. CA Cancer J Clin.

[2] Sun W, Yuan X, Tian Y, Wu H, Xu H, Hu G, Wu K (2015). Non-invasive approaches to monitor EGFR-TKI treatment in non-small-cell lung cancer. J Hematol Oncol.

[3] Schiller JH, Harrington D, Belani CP, Langer C, Sandler A, Krook J, Zhu J, Johnson DH (2002). Eastern Cooperative Oncology Group. Comparison of four chemotherapy regimens for advanced non-small-cell lung cancer. N Engl J Med.

[4] Kanzawa F, Matsushima Y, Nakano H, Nakagawa K, Takahashi H, Sasaki Y, Saijo N (1988). Antitumor activity of a new platinum compound (glycolate-o,o’) diammineplatinum (II) (254-S), against non-small cell lung carcinoma grown in a human tumor clonogenic assay system. Anticancer Res.

[5] Naito Y, Kubota K, Ohmatsu H, Goto K, Niho S, Yoh K, Ohe Y (2011). Phase II study of nedaplatin and docetaxel in patients with advanced squamous cell carcinoma of the lung. Ann Oncol.

[6] Sekine I, Nokihara H, Horiike A, Yamamoto N, Kunitoh H, Ohe Y, Tamura T, Kodama T, Saijo N (2004). Phase I study of cisplatin analogue nedaplatin (254-S) and paclitaxel in patients with unresectable squamous cell carcinoma. Br J Cancer.

[7] Oshita F, Yamada K, Kato Y, Ikehara M, Noda K, Tanaka G, Nomura I, Suzuki R, Saito H (2003). Phase I/II study of escalating doses of nedaplatin in combination with irinotecan for advanced non-small-cell lung cancer. Cancer Chemother Pharmacol.

[8] Kurata T, Tamura K, Yamamoto N, Nogami T, Satoh T, Kaneda H, Nakagawa K, Fukuoka M (2004). Combination phase I study of nedaplatin and gemcitabine for advanced non-small-cell lung cancer. Br J Cancer.

[9] Oshita F, Yamada K, Saito H, Noda K, Hamanaka N, Ikehara M (2004). Phase II study of nedaplatin and irinotecan for elderly patients with advanced non-small cell lung cancer. J Exp Ther Oncol.

[10] Shirai T, Hirose T, Noda M, Ando K, Ishida H, Hosaka T, Ozawa T, Okuda K, Ohnishi T, Ohmori T, Horichi N, Adachi M (2006). Phase II study of the combination of gemcitabine and nedaplatin for advanced non-small-cell lung cancer. Lung Cancer.

[11] Oshita F, Honda T, Murakami S, Kondo T, Saito H, Noda K, Yamada K (2011). Comparison of nedaplatin and irinotecan for patients with squamous and nonsquamous cell carcinoma of the lung: meta-analysis of four trials. J Thorac Oncol.

[12] Yamamoto N, Tamura T, Kurata T, Yamamoto N, Sekine I, Kunitoh H, Ohe Y, Saijo N (2009). A dose-finding and pharmacokinetic study of nedaplatin in elderly patients with advanced non-small cell lung cancer. Cancer Chemother Pharmacol.

[13] Teramoto K, Asada Y, Ozaki Y, Suzumura Y, Nakano Y, Sawai S, Tezuka N, Inoue S, Fujino S (2012). A phase II study of docetaxel plus nedaplatin in patients with metastatic non-small-cell lung cancer. Cancer Chemother Pharmacol.

[14] Yang JJ, Zhou Q, Liao RQ, Huang YS, Xu CR, Wang Z, Wang BC, Chen HJ, Wu YL (2012). Nedaplatin/Gemcitabine Versus Carboplatin/Gemcitabine in Treatment of Advanced Non-small Cell Lung Cancer: A Randomized Clinical Trial. Chin J Cancer Res.

[15] Li CH, Liu MY, Liu W, Li DD, Cai L (2014). Randomized Control Study of Nedaplatin or Cisplatin Concomitant with Other Chemotherapy in the Treatment of Advanced Non-small Cell Lung Cancer. Asian Pac J Cancer Prev.

[16] Zhang K, Qin H, Pan F, Liu E, Liang H, Ruan Z (2014). Nedaplatin or oxaliplatin combined with paclitaxel and docetaxel as first-line treatment for patients with advanced non-small cell lung cancer. Med Sci Monit.

[17] Fukuda M, Shinkai T, Eguchi K, Sasaki Y, Tamura T, Ohe Y, Kojima A, Oshita F, Hara K, Saijo N (1990). Phase II study of (glycolate-O,O’) diammineplatinum(II), a novel platinum complex, in the treatment of non-small-cell lung cancer. Cancer Chemother Pharmacol.

[18] Sekine I, Sumi M, Ito Y, Kato T, Fujisaka Y, Nokihara H, Yamamoto N, Kunitoh H, Ohe Y, Tamura T (2007). Phase I study of cisplatin analogue nedaplatin, paclitaxel, and thoracic radiotherapy for unresectable stage III non-small cell lung cancer. Jpn J Clin Oncol.

[19] Yamada K, Saito H, Kondo T, Murakami S, Masuda N, Yamamoto M, Igawa S, Katono K, Takiguchi Y, Iwasawa S, Kurimoto R, Okamoto H, Shimokawa T (2015). Multicenter Phase II Study of Nedaplatin and Irinotecan for Patients with Squamous Cell Carcinoma of the Lung: Thoracic Oncology Research Group 0910. Anticancer Res.

[20] Shukuya T, Yamanaka T, Seto T, Daga H, Goto K, Saka H, Sugawara S, Takahashi T, Yokota S, Kaneda H, Kawaguchi T, Nagase S, Oguri T (2015). Nedaplatin plus docetaxel versus cisplatin plus docetaxel for advanced or relapsed squamous cell carcinoma of the lung (WJOG5208L): a randomised, open-label, phase 3 trial. Lancet Oncol.

[21] Masago K, Fujita S, Kim YH, Hatachi Y, Fukuhara A, Irisa K, Nagai H, Sakamori Y, Togashi Y, Mio T, Mishima M (2011). Phase I study of the combination of nedaplatin and gemcitabine in previously untreated advanced squamous cell lung cancer. Cancer Chemother Pharmacol.

[22] Hirose T, Horichi N, Ohmori T, Shirai T, Sohma S, Yamaoka T, Ohnishi T, Adachi M (2003). Phase I study of the combination of gemcitabine and nedaplatin for treatment of previously untreated advanced non-small cell lung cancer. Lung Cancer.

[23] Tang Y, Wang W, Teng XZ, Shi L (2014). Efficacy of S-1 plus nedaplatin compared to standard second-line chemotherapy in EGFR-negative lung adenocarcinoma after failure of first-line chemotherapy. Tumour Biol.

[24] Desoize B, Madoulet C (2002). Particular aspects of platinum compounds used at present in cancer treatment. Crit Rev Oncol Hematol.

[25] Nakamura Y, Hasegawa M, Hayakawa K, Matsuura M, Suzuki Y, Nasu S, Yamakawa M, Mitsuhashi N, Niibe H (2000). Induction of p53-dependent apoptosis *in vivo* by nedaplatin and ionizing radiation. Oncol Rep.

[26] Tian Y, Wu K, Liu Q, Han N, Zhang L, Chu Q, Chen Y (2016). Modification of platinum sensitivity by KEAP1/NRF2 signals in non-small cell lung cancer. J Hematol Oncol.

[27] Tian Y, Liu Q, He X, Yuan X, Chen Y, Chu Q, Wu K (2016). Emerging roles of Nrf2 signal in non-small cell lung cancer. J Hematol Oncol.

[28] Krall EB, Wang B, Munoz DM, Ilic N, Raghavan S, Niederst MJ, Yu K, Ruddy DA, Aguirre AJ, Kim JW, Redig AJ, Gainor JF, Williams JA (2017). KEAP1 loss modulates sensitivity to kinase targeted therapy in lung cancer. eLife.

[29] Kanzawa F, Koizumi F, Koh Y, Nakamura T, Tatsumi Y, Fukumoto H, Saijo N, Yoshioka T, Nishio K (2001). *In vitro* synergistic interactions between the cisplatin analogue nedaplatin and the DNA topoisomerase I inhibitor irinotecan and the mechanism of this interaction. Clin Cancer Res.

[30] Yamada H, Uchida N, Maekawa R, Yoshioka T (2001). Sequence-dependent antitumor efficacy of combination chemotherapy with nedaplatin, a newly developed platinum, and paclitaxel. Cancer Lett.

[31] Liu Y, Yu S, Liu S, Cao H, Ma R, Wu J, Feng J (2015). Comparison of nedaplatin-based versus cisplatin-based chemotherapy for advanced non-small cell lung cancer among East Asian populations: A meta-analysis. Sci Rep.

